# Supplementary material for: Exercise across the Lung Cancer Care Continuum: An Overview of Systematic Reviews
Source: J Clin Med. 2023 Feb 27;12(5):1871. doi: 10.3390/jcm12051871 (PMC10003899; doi:10.3390/jcm12051871)
Supplement: Supplementary file 1 [file jcm-12-01871-s001.zip › jcm-2202180-supplementary.pdf]

Table S1. Overview PICO research question criteria

Population	Intervention	Comparator	Outcomes	Setting
• Adults (≥18yo)<br>• Lung Cancer<br>• With or without metastasis<br>• Any stage<br>• Receiving any curative or palliative treatments, or no treatment at all	One or more of the following exercise interventions<br>• Aerobic exercise<br>• Resistance exercise<br>• Stretching, balance, yoga or tai chi<br>• Respiratory exercise, including inspiratory muscle training<br>• Electrical stimulation<br>• Education or behavioural interventions aimed at increasing physical activity<br>May be supervised or unsupervised	Usual care or any non-exercise intervention	At least one health-related outcome.<br><br>Primary overview outcomes included<br>• Exercise capacity<br>• Physical function<br>• Health-related quality of life<br>• Post-operative pulmonary complications	All settings where exercise is delivered<br>• Hospital inpatient<br>• Outpatient clinics<br>• Community centre or gym<br>• Home-based<br>• Telehealth

Table S2. Database search strategies

MEDLINE (PubMed) search strategy

Set	Search Statement
1.	exp Carcinoma, Small Cell/ or Carcinoma, Small Cell.tw. or (exp Carcinoma, Non-Small-Cell Lung/ or Carcinoma, Non-Small-Cell Lung.tw.) or (exp Lung Neoplasms/ or Lung Neoplasms.tw.)
2.	(lung cancer* or lung carcinoma* or lung malignan* or lung tumo* or pulmonary cancer* or pulmonary carcinom* or pulmonary malignan* or pulmonary neoplasm* or pulmonary tumo* or Non Small Cell* or Nonsmall cell* or Non-small cell* or Non-small-cell* or NSCLC or SCLC or small cell* or small-cell or oat cell lung carcinoma* or oat cell cancer* or oat cell lung cancer* or oat cell carcinoma*).tw.
3.	1 or 2
4.	exp Exercise/ or Exercise.tw. or (exp Exercise Therapy/ or Exercise Therapy.tw.) or (exp Preoperative Exercise/ or Preoperative Exercise.tw.) or (exp Physical Endurance/ or Physical Endurance.tw.) or (exp Exercise Movement Techniques/ or Exercise Movement Techniques.tw.) or (exp Respiratory Muscles/ or Respiratory Muscle*.tw.) or (exp Bicycling/ or Bicycling.tw.) or (exp Rehabilitation/ or Rehabilitation.tw.) or (exp Telerehabilitation/ or Telerehabilitation.tw.) or (exp walking/ or walking.tw.) or (exp resistance training/ or resistance training.tw.) or (exp Physical Therapy Modalities/ or Physical Therapy Modalities.tw.) or (breathing exercise/ or breathing exercise*.tw.)
5.	(Exercis* or aerobic* or isometric or eccentric or physical conditioning or walk* or treadmill* or running or jogging or swimming or high intensity interval or HIIT or warm up or cool down or yoga or pilates or tai chi or taichi or motion therap* or stretching or plyometric or exercise rehabilitation or Physical activity or physical endurance or endurance or strength* or resistance training or weight training or weight lifting or bicyc* or cycling or tai ji or taiji or dance therapy or preoperative exercise* or pre operative exercise* or preoperative rehabilitat* or pre operative rehabilitat* or preoperative condit* or pre operative condit* or prehab* or pre hab* or rehabilitat* or telerehabilitat* or tele rehabilitat* or remote rehabilitat* or virtual rehabilitat* or breathing exercise* or inspiratory muscle* or expiratory muscle* or physical therap* or physiother*).tw.

6.	4 or 5
7.	(Systematic review or review).af.
8.	3 and 6 and 7
9.	exp animals/
10.	humans.sh.
11.	9 Nt 10
12.	8 Nt 11

Embase Ovid search strategy

1.	exp lung tumor/ or exp non small cell lung cancer/ or small cell lung cancer/
2.	(lung cancer* or lung carcinoma* or lung malignan* or lung tumo* or pulmonary cancer* or pulmonary carcinom* or pulmonary malignan* or pulmonary neoplasm* or pulmonary tumo* or Non Small Cell* or Nonsmall cell* or Non-small cell* or Non-small-cell* or NSCLC or SCLC or small cell* or small-cell or oat cell).ti,ab.
3.	1 or 2
4.	exp exercise/ or exp kinesiotherapy/ or exp endurance/ or exp breathing muscles/ or exp breathing exercise/ or exp rehabilitation/ or exp walking/ or exp resistance training/ or exp cycling/ or exp aerobic exercise/ or exp telerehabilitation/
5.	(Exercis* or aerobic* or isometric or eccentric or physical conditioning or walking or treadmill* or running or jogging or swimming or high intensity interval or HIIT or warm up or cool down or yoga or pilates or tai chi or taichi or motion therap* or stretching or plyometric or exercise rehabilitation or Physical activity or physical endurance or endurance or strength* or resistance training or weight training or weight lifting or bicyc* or cycling or tai ji or taiji or dance therapy or preoperative exercise* or pre operative exercise* or preoperative rehabilitat* or pre operative rehabilitat* or preoperative condit* or pre operative condit* or prehab* or pre hab* or rehabilitat* or telerehabilitat* or tele rehabilitat* or remote rehabilitat* or virtual rehabilitat* or breathing exercise* or inspiratory muscle* or expiratory muscle* or physical therap* or physiother*).ti,ab.
6.	4 or 5
7.	(Review or Systematic Review).af.
8.	3 and 6 and 7
9.	exp animal/ or exp invertebrate/ or Nonhuman/ or animal experiment/ or animal tissue/ or animal model/ or exp plant/ or exp fungus/
12.	8 Not 9

Cochrane CENTRAL and CDRS search strategy

#1 MeSH descriptor: [Carcinoma, Non-Small-Cell Lung] explode all trees
#2 MeSH descriptor: [Carcinoma, Small Cell] explode all trees
#3 MeSH descriptor: [Lung Neoplasms] explode all trees
#4 #1 or #2 or #3
#5 ("lung cancer*" or "lung carcinoma*" or "lung malignan*" or "lung tumo*" or "pulmonary cancer*" or "pulmonary carcinom*" or "pulmonary malignan*" or "pulmonary neoplasm*" or "pulmonary tumo*" or "Non Small Cell*" or "Nonsmall cell*" or "Non-small cell*" or "Non-small-cell*" or NSCLC or SCLC or "small cell*" or "small-cell" or "oat cell*"):ti OR

("lung cancer*" or "lung carcinoma*" or "lung malignan*" or "lung tumo*" or "pulmonary cancer*" or "pulmonary carcinom*" or "pulmonary malignan*" or "pulmonary neoplasm*" or "pulmonary tumo*" or "Non Small Cell*" or "Nonsmall cell*" or "Non-small cell*" or "Non-small-cell*" or NSCLC or SCLC or "small cell*" or "small-cell" or "oat cell*"):ab
#6 #4 or #5
#7 MeSH descriptor: [Exercise] explode all trees
#8 MeSH descriptor: [Exercise Therapy] explode all trees
#9 MeSH descriptor: [Preoperative Exercise] explode all trees
#10 MeSH descriptor: [Physical Endurance] explode all trees
#11 MeSH descriptor: [Exercise Movement Techniques] explode all trees
#12 MeSH descriptor: [Respiratory Muscles] explode all trees
#13 MeSH descriptor: [Bicycling] explode all trees
#14 MeSH descriptor: [Rehabilitation] explode all trees
#15 MeSH descriptor: [Telerehabilitation] explode all trees
#16 MeSH descriptor: [Walking] explode all trees
#17 MeSH descriptor: [Resistance Training] explode all trees
#18 MeSH descriptor: [Physical Therapy Modalities] explode all trees
#19 MeSH descriptor: [Breathing Exercises] explode all trees
#20 (Exercis* or aerobic* or isometric or eccentric or "physical conditioning " or walking or treadmill* or running or jogging or swimming or "high intensity interval" or HIIT or "warm up" or "cool down" or yoga or pilates or "tai chi" or taichi or "motion therap*" or stretching or plyometric or "exercise rehabilitation" or "Physical activity" or "physical endurance" or endurance or strength* or "resistance training" or "weight training" or "weight lifting" or bicyc* or cycling or tai ji or taiji or dance therapy or preoperative exercise* or pre operative exercise* or "preoperative rehabilitat*" or "pre operative rehabilitat*" or "preoperative condit*" or "pre operative condit*" or prehab* or "pre hab*" or rehabilitat* or telerehabilitat* or "tele rehabilitat*" or "remote rehabilitat*" or "virtual rehabilitat*" or "inspiratory muscle*" or "expiratory muscle*" or "physical therap*" or physiother*):ti OR (Exercis* or aerobic* or isometric or eccentric or "physical conditioning " or walking or treadmill* or running or jogging or swimming or "high intensity interval" or HIIT or "warm up" or "cool down" or yoga or pilates or "tai chi" or taichi or "motion therap*" or stretching or plyometric or "exercise rehabilitation" or "Physical activity" or "physical endurance" or endurance or strength* or "resistance training" or "weight training" or "weight lifting" or bicyc* or cycling or tai ji or taiji or dance therapy or preoperative exercise* or pre operative exercise* or "preoperative rehabilitat*" or "pre operative rehabilitat*" or "preoperative condit*" or "pre operative condit*" or prehab* or "pre hab*" or rehabilitat* or telerehabilitat* or "tele rehabilitat*" or "remote rehabilitat*" or "virtual rehabilitat*" or "inspiratory muscle*" or "expiratory muscle*" or "physical therap*" or physiother*):ab
#21 #7 or #8 or #9 or #10 or #11 or #12 or #13 or #14 or #15 or #16 or #17 or #18 or #19 or #20
#22 (systematic review or review)
#23 #6 and #21 AND #22

DARE search strategy

Set	Search Statement
1.	(Lun neoplasm* or lung cancer* or lung carcinoma* or lung malignan* or lung tumo* or pulmonary cancer* or pulmonary carcinom* or pulmonary malignan* or pulmonary neoplasm* or pulmonary tumo* or Non Small Cell* or Nonsmall cell* or Non-small cell* or Non-small-cell* or NSCLC or SCLC or small cell* or small-cell or oat cell lung carcinoma* or oat cell cancer* or oat cell lung cancer* or oat cell carcinoma*).tw.
2.	(Exercis* or aerobic* or isometric or eccentric or physical conditioning or walk* or treadmill* or running or jogging or swimming or high intensity interval or HIIT or warm up or cool down or yoga or pilates or tai chi or taichi or motion therap* or stretching or plyometric or exercise rehabilitation or Physical activity or physical endurance or endurance or strength* or resistance training or weight training or weight lifting or bicyc* or cycling or tai ji or taiji or dance therapy or preoperative exercise* or pre operative exercise* or preoperative rehabilitat* or pre operative rehabilitat* or preoperative condit* or pre operative condit* or prehab* or pre hab* or rehabilitat* or telerehabilitat* or tele rehabilitat* or tele-rehabiltat*or remote rehabilitat* or virtual rehabilitat* or breathing exercise* or inspiratory muscle* or expiratory muscle* or physical therap* or physiother* or respiratory muscle* or rehabilitation or walking or breathing exercise* or breathing muscle*).tw.
3.	(Systematic review or review).af.

4.	1 and 2 and 3
5.	animal*.tw.
7.	5 Not 4

CINAHL and sportdiscus search strategy

S1	((MH "Carcinoma, Non-Small-Cell Lung+") OR (MH "Carcinoma, Small Cell+") OR (MH "Lung Neoplasms+")) OR TI (("lung cancer*" or "lung neoplasm" or "lung carcinoma*" or "lung malignan*" or "lung tumo*" or "pulmonary cancer*" or "pulmonary carciNm*" or "pulmonary malignan*" or "pulmonary neoplasm*" or "pulmonary tumo*" or "Non Small Cell*" or "Nonsmall cell*" or "Non-small cell*" or "Non-small-cell*" or NSCLC or SCLC or "small cell*" or small-cell or "oat cell")) OR AB (("lung cancer*" or "lung neoplasm" or "lung carciNma*" or "lung malignan*" or "lung tumo*" or "pulmonary cancer*" or "pulmonary carcinom*" or "pulmonary malignan*" or "pulmonary neoplasm*" or "pulmonary tumo*" or "Non Small Cell*" or "Nonsmall cell*" or "Non-small cell*" or "Non-small-cell*" or NSCLC or SCLC or "small cell*" or small-cell or "oat cell"))
S2	((MH "Exercise+") OR (MH "Resistance Training+") OR (MH "Therapeutic Exercise+") OR (MH "Prehabilitation+") OR (MH "Respiratory Muscles+") OR (MH "Cycling+") OR (MH "Rehabilitation+") OR (MH "Telerehabilitation+") OR (MH "Breathing Exercises+") or (MH "Walking+") OR (MH "Aerobic exercise+") OR (MH "Physical Therapy+")) OR TI ((Exercis* or aerobic* or isometric or eccentric or "physical conditioning" or walking or treadmill* or running or jogging or swimming or "high intensity interval" or HIIT or "warm up" or "cool down" or yoga or pilates or "tai chi" or taichi or "motion therap*" or stretching or plyometric or "exercise rehabilitation" or "Physical activity" or "physical endurance" or endurance or strength* or "resistance training" or "weight training" or "weight lifting" or bicyc* or cycling or "tai ji" or taiji or "dance therapy" or preoperative exercise* or pre operative exercise* or "preoperative rehabilitat*" or "pre operative rehabilitat*" or "preoperative condit*" or "pre operative condit*" or prehab* or "pre hab*" or rehabilitat* or telerehabilitat* or "tele rehabilitat*" or "remote rehabilitat*" or "virtual rehabilitat*" or "inspiratory muscle*" or "expiratory muscle*" or "physical therap*" or physiother* or breathing exercise*)) OR AB ((Exercis* or aerobic* or isometric or eccentric or "physical conditioning" or walking or treadmill* or running or jogging or swimming or "high intensity interval" or HIIT or "warm up" or "cool down" or yoga or pilates or "tai chi" or taichi or "motion therap*" or stretching or plyometric or "exercise rehabilitation" or "Physical activity" or "physical endurance" or endurance or strength* or "resistance training" or "weight training" or "weight lifting" or bicyc* or cycling or "tai ji" or taiji or "dance therapy" or preoperative exercise* or pre operative exercise* or "preoperative rehabilitat*" or "pre operative rehabilitat*" or "preoperative condit*" or "pre operative condit*" or prehab* or "pre hab*" or rehabilitat* or telerehabilitat* or "tele rehabilitat*" or "remote rehabilitat*" or "virtual rehabilitat*" or "inspiratory muscle*" or "expiratory muscle*" or "physical therap*" or physiother* or breathing exercise*))
S3	TX "systematic review" or review
S4	((MH "Animals+") OR (MH "Animal Studies") OR (MH "animal model*")) NT (MH "human")
S5	S1 AND S2 AND S3
S6	S5 NOT S4

PEDro search strategy

"Lung Cancer"

Table S3. Excluded full-text references and reasons for exclusion

	Study	Reason for exclusion
1	Archer & Ciechanowicz, 2019 [1]	Conference abstract. Unable to retrieve report for the full study.
2	Barakat et al., 2018 [2]	Wrong population. Data for lung cancer patients is not reported separately.
3	Bayly et al., 2018 [3]	Wrong population. Data for lung cancer patients is not reported separately. Data for RCTs not synthesised separately.
4	Bibo, Goldblatt, & Merry, 2021 [4]	Wrong study design. Not a Systematic Review.
5	Castro, Codima, Das Neves, & Borges, 2019 [5]	Conference abstract. The published study included in review.
6	Chan et al., 2012 [6]	Wrong included studies. Data for RCTs not synthesised separately.
7	Clark, Maguire, Cannon, & Leung, 2021 [7]	Wrong population. Data for lung cancer patients is not reported separately.
8	Crandall, Kearney, Maguire, & Campbell, 2014 [8]	Wrong included studies. Data for RCTs not synthesised separately.
9	Curry, Patterson, Greenley, Pearson, & Forbes, 2021 [9]	Wrong comparator.
10	Devor et al., 2013 [10]	Wrong population. <50% lung cancer population in primary studies. Lung cancer not reported separately.
11	Dittus, Ades, & Gramling, 2017 [11]	Wrong population. Data for lung cancer patients is not reported separately.
12	dos Santos Goes & Santos do Nascimento, 2021 [12]	Wrong study design. Not a Systematic Review.
13	Driessen et al., 2017 [13]	Wrong population. Data for lung cancer patients is not reported separately.
14	Du et al., 2015 [14]	Wrong population. Data for lung cancer patients is not reported separately.
15	Dy et al., 2020 [15]	Wrong population. Data for lung cancer patients is not reported separately.
16	Easwaran et al., 2021 [16]	Wrong population. <50% lung cancer population in primary studies.
17	Ester et al., 2021 [17]	Wrong population. <50% lung cancer population in primary studies. Does not report separately for lung cancer.
18	Faithfull et al., 2019 [18]	Wrong population. <50% lung cancer population in primary studies. Lung cancer is not reported separately.
19	Ferreira et al., 2021 [19]	Wrong intervention
20	García & Yáñez Brage, 2013 [20]	Language. Not written in English.
21	Grande et al., 2021 [21]	Wrong population. <50% lung cancer population in primary studies.
22	Granger, Denehy, McDonald, Berney, & Chao, 2011 [22]	Wrong included studies. Data for RCTs not synthesised separately.
23	Groll, Tranmer, Woodend, & Luctkar-Flude, 2007 [23]	Wrong population. Data for lung cancer patients is not reported separately.
24	Henshall, Allin, & Aveyard, 2019 [24]	Wrong population. Data for lung cancer patients is not reported separately.<br>Wrong included studies. Data for RCTs not synthesised separately.
25	Heywood, McCarthy, Skinner, McCarthy, & Skinner, 2017 [25]	Wrong population. Data for lung cancer patients is not reported separately.<br>Wrong included studies. Data for RCTs not synthesised separately.
26	Heywood, Skinner, & McCarthy, 2018 [26]	Wrong population. Data for lung cancer patients is not reported separately.<br>Wrong included studies. Data for RCTs not synthesised separately.

27	Huang, Peng, Cheng, Huang, & Chang, 2021 [27]	Wrong comparator
28	Johnson & Currow, 2016 [28]	Wrong study design. Not a systematic review.
29	Kalter et al., 2017 [29]	Wrong population. <50% lung cancer population in primary studies. Lung cancer not reported separately.
30	Kasparov et al., 2019 [30]	Conference abstract.
31	Koeppel, Mathis, Schmitz, & Wiskemann, 2021 [31]	Wrong population. <50% lung cancer population in primary studies.
32	Kuo et al., 2021 [32]	Wrong population. <50% lung cancer population in primary studies. Lung cancer not reported separately.
33	R. Li et al., 2021 [33]	Wrong included studies. Data for RCTs not synthesised separately.
34	W. Liu et al., 2013 [34]	Data for RCTs not synthesised separately.
35	Lonbro, 2014 [35]	Data for lung cancer patients is not reported separately.
36	Lowe, Watanabe, & Courneya, 2009 [36]	Data for lung cancer patients is not reported separately.<br>Data for RCTs not synthesised separately.
37	Ma, Tan, Yang, Liao, & Huang, 2016 [37]	Data for lung cancer patients is not reported separately.
38	Makwana, Makwana, & Shetye, 2016 [38]	Data for RCTs not synthesised separately.
39	Mao, Ni, Niu, & Jiang, 2021 [39]	Wrong study design
40	Marthick et al., 2021 [40]	Wrong population
41	Medysky et al., 2021 [41]	Incorrect outcomes collected.<br>Principles of exercise prescription rather than patient outcomes.
42	Meneses-Echavez, Loaiza-Betancur, Triana-Reina, Diaz-Lopez, & Echavarria-Rodriguez, 2021 [42]	Wrong population
43	Michael, Lehrer, Schmitz, & Zaorsky, 2021 [43]	Wrong population
44	Nan et al., 2018 [44]	Conference abstract.
45	Ni et al., 2017 [45]	Wrong included studies. Data for RCTs not synthesised separately.
46	Paramanandam & Dunn, 2013 [46]	Conference abstract only.
47	Paramanandam & Dunn, 2015 [47]	Wrong included studies. Data for RCTs not synthesised separately.
48	Piraux, Caty, & Reychler, 2018 [48]	Wrong population. Data for lung cancer patients is not reported separately.
49	Pouwels et al., 2015 [49]	Wrong included studies. Data for RCTs not synthesised separately.
50	Rathore & Afridi, 2021 [50]	Wrong study design
51	Rowntree & Hosseinzadeh, 2022 [51]	Wrong intervention
52	Ream et al., 2020 [52]	Wrong population. <50% lung cancer population in primary studies.
53	Rodriguez-Larrad, Lascurain-Aguirrebena, Abecia-Inchaurregui, & Seco, 2014 [53]	Wrong Control. Control group did not exclusively receive usual care or non-exercise intervention.
54	Sanchez-Lorente et al., 2018 [54]	Wrong study design. Not a systematic review.
55	Sebio Garcia, Yanez Brage, Gimenez Moolhuyzen, Granger, & Denehy, 2016 [55]	Data for RCTs not synthesised separately.
56	F. Singh, Newton, Galvao, Spry, & Baker, 2013 [56]	Data for lung cancer patients is not reported separately.<br>Data for RCTs not synthesised separately.
57	S. Song et al., 2018 [57]	Does not synthesise for lung cancer with control group of usual care or non-exercise intervention.

58	Y. Song et al., 2020 [58]	Data for lung cancer patients is not reported separately.
59	Stoner, Hanson, Khosravi, & Farajivafa, 2019 [59]	Only one primary study included patients with lung cancer.
60	Tahirah, Cavalheri, Hill, Nonoyama, & Jenkins, 2014 [60]	This systematic review has been updated.
61	Thompson, Sola, & Subirana, 2005 [61]	Only one primary study included exercise as an intervention.
62	Turner et al., 2018 [62]	Data for lung cancer patients is not reported separately.<br>Limited lung cancer population in primary studies.
63	Y.-Q. Wang et al., 2019 [63]	Control group did not exclusively receive usual care or non-exercise intervention.
64	Yorke, Brettle, & Molassiotis, 2012 [64]	Data for exercise interventions is not reported separately.
65	I. Zhao & Yates, 2008 [65]	Data for RCTs not synthesised separately.
66	Y. Zhao, Zheng, Xiang, Ning, & Li, 2021 [66]	Wrong population
67	Liang Zhou, Qijiu Chen, & Jianyong Zhang, 2021 [67]	Wrong Control. Control group did not exclusively receive usual care or non-exercise intervention.

Table S4. AMSTAR-2 items and questions

Item	Question
1	Did the research questions and inclusion criteria for the review include the components of PICO?
2	Did the report of the review contain an explicit statement that the review methods were established prior to the conduct of the review and did the report justify any significant deviations from the protocol?
3	Did the review authors explain their selection of the study designs for inclusion in the review?
4	Did the review authors use a comprehensive literature search strategy?
5	Did the review authors perform study selection in duplicate?
6	Did the review authors perform data extraction in duplicate?
7	Did the review authors provide a list of excluded studies and justify the exclusions?
8	Did the review authors describe the included studies in adequate detail?
9	Did the review authors use a satisfactory technique for assessing the RoB in individual studies that were included in the review?
10	Did the review authors report on the sources of funding for the studies included in the review?
11	If meta-analysis was performed did the review authors use appropriate methods for statistical combination of results?
12	If meta-analysis was performed, did the review authors assess the potential impact of RoB in individual studies on the results of the meta-analysis or other evidence synthesis?
13	Did the review authors account for RoB in individual studies when interpreting/ discussing the results of the review?
14	Did the review authors provide a satisfactory explanation for, and discussion of, any heterogeneity observed in the results of the review?
15	If they performed quantitative synthesis did the review authors carry out an adequate investigation of publication bias (small study bias) and discuss its likely impact on the results of the review?
16	Did the review authors report any potential sources of conflict of interest, including any funding they received for conducting the review?

Footnote:RoB=risk of bias

Table S5. AMSTAR-2 methodological quality ratings

Item	Rueda 2011	Mainini 2016	Hsieh 2017	Li 2017	Cavalheri 2017	Steffens 2018	Traenor 2018	Sommer 2018	Papadopolous 2018	Larsen 2018	Wang 2018	Rosero 2019	Peddle McIntyre 2019	Cavalheri 2019	Liu 2019	Li 2019	Ma 2020	Himbert 2020	Singh 2020	Yang 2020	Codima 2021	Ma 2021	Lee 2021	Pu 2021	Gravier 2021	Heredia-Ciuro 2021	Machado 2021	Zhou 2021	De Oliveira Vacchi 2022	Xu 2022
1	N	N	Y	Y	Y	Y	Y	Y	N	Y	N	N	Y	Y	N	Y	Y	N	Y	Y	Y	Y	Y	Y	Y	Y	Y	N	N	Y
2	Y	N	N	N	Y	PY	PY	PY	N	Y	N	N	Y	Y	N	PY	N	N	N	N	PY	PY	N	N	Y	PY	PY	N	PY	N
3	N	Y	N	N	Y	Y	N	Y	N	N	Y	N	N	N	N	N	Y	N	N	Y	N	Y	N	Y	Y	N	N	Y	Y	N
4	N	PY	PY	PY	Y	Y	PY	Y	PY	Y	PY	PY	Y	Y	N	PY	PY	N	PY	PY	PY	PY	PY	PY	PY	PY	PY	PY	PY	PY
5	Y	Y	N	N	Y	N	Y	Y	Y	Y	Y	Y	Y	Y	N	N	Y	Y	N	N	Y	Y	Y	Y	Y	Y	Y	Y	Y	N
6	N	Y	Y	Y	Y	Y	Y	Y	Y	Y	N	Y	Y	Y	Y	Y	N	Y	N	Y	N	Y	N	Y	Y	N	Y	Y	Y	N
7	Y	Y	N	N	Y	N	N	N	N	Y	N	Y	Y	Y	N	Y	N	N	N	N	Y	N	N	N	N	N	N	N	N	N
8	Y	Y	PY	Y	Y	PY	Y	Y	Y	Y	N	PY	Y	Y	PY	PY	Y	PY	PY	Y	PY	PY	PY	PY	Y	Y	Y	Y	Y	Y
9	Y	Y	Y	Y	Y	Y	Y	Y	Y	Y	Y	Y	Y	Y	Y	Y	Y	Y	Y	Y	Y	Y	Y	Y	Y	Y	Y	Y	Y	Y
10	N	N	N	N	N	N	N	N	N	N	N	N	N	N	N	N	N	N	N	N	N	N	N	N	N	N	N	N	N	N
11	NA	NA	Y	Y	Y	Y	Y	Y	Y	Y	Y	N	Y	Y	Y	Y	NA	NA	Y	Y	NA	Y	N	Y	Y	N	Y	Y	Y	Y
12	NA	NA	Y	Y	N	N	N	N	Y	Y	Y	N	N	Y	Y	N	NA	NA	Y	Y	NA	N	N	N	Y	N	Y	Y	N	N
13	Y	Y	N	Y	Y	N	N	Y	Y	N	N	N	Y	Y	N	N	N	N	N	N	Y	N	N	N	Y	N	N	N	N	N
14	N	N	N	Y	Y	Y	Y	N	Y	Y	Y	N	Y	Y	N	N	N	N	N	Y	N	Y	N	Y	Y	N	Y	Y	Y	Y
15	NA	NA	N	Y	N	N	N	N	Y	N	Y	Y	N	N	Y	Y	NA	NA	N	Y	NA	Y	N	N	Y	N	Y	N	N	Y
16	Y	Y	Y	Y	Y	Y	Y	Y	Y	Y	Y	Y	Y	Y	N	Y	N	Y	Y	Y	Y	Y	Y	N	Y	Y	Y	Y	Y	Y
CW (N)	1	1	4	2	1	3	3	2	2	2	3	3	1	1	4	1	3	4	4	3	0	2	5	4	1	4	2	4	3	3
N-CW (N)	5	3	4	3	2	3	3	3	3	2	4	5	3	2	6	5	4	4	5	2	4	2	5	3	0	5	2	2	3	5
Overall rating	L	L	CL	CL	L	CL	CL	CL	CL	CL	CL	CL	L	L	CL	L	CL	CL	CL	CL	M	CL	CL	CL	L	CL	CL	CL	CL	CL

Footnote: AMSTAR-2 ratings: Y=yes, N=no, PY=partial yes, NA=not applicable; Overall rating: M=moderate, L=low, CL=critically low. Abbreviations: CW=critical weakness; N-CW=non-critical weakness.

Table S6. PRISMA reporting items

PRISMA 2020 Item	
**Title**	
1	Title
**Abstract**	
2 i	Title
2 ii	Objectives
2 iii	Eligibility criteria
2 iv	Information sources
2 v	Risk of bias
2 vi	Synthesis of results
2 vii	Included studies
2 viii	Synthesis of results
2 ix	Limitations of evidence
2 x	Interpretation
2 xi	Funding
2 xii	Registration
**Introduction**	
3	Rationale
4	Objectives
**Methods**	
5	Eligibility criteria
6	Information sources
7	Search strategy
8	Selection process
9	Data collection process
10a	Data Items - outcomes
10b	Data Items – other variables
11	Study risk of bias assessment
12	Effect measures
13a	Synthesis methods - eligibility

13b	Synthesis methods – data preparation
13c	Synthesis methods – tables/figs
13d	Synthesis methods - rationale
13e	Synthesis methods - heterogeneity
13f	Synthesis methods - sensitivity
14	Reporting bias assessment
15	Certainty assessment
**Results**	
16a	Study selection – results (flow)
16b	Study selection - excluded
17	Study characteristics
18	Risk of bias in studies
19	Results of individual studies
20a	Results of syntheses – study characteristics and risk of bias
20b	Results of syntheses - all statistical syntheses
20c	Results of syntheses – heterogeneity
20d	Results of syntheses – sensitivity
21	Reporting biases
22	Certainty of evidence
**Discussion**	
23a	Discussion - interpretation
23b	Discussion – evidence limitations
23c	Discussion – review limitations
23d	Discussion - implications
**Other Information**	
24a	Registration and protocol – registration details
24b	Registration and protocol –protocol access
24c	Registration and protocol – amendments
25	Support – review funding
26	Competing interests
27	Availability of data, code and other materials

Table S7. PRISMA reporting ratings

Item	Systematic review	Rueda 2011	Mainini 2016	Hsieh 2017	Li 2017	Cavalheri 2017	Steffens 2018	Traenor 2018	Sommer 2018	Papadopolous 2018	Larsen 2018	Wang 2018	Rosero 2018	Peddle McIntyre 2019	Cavalheri 2019	Liu 2019	Li 2019	Ma 2020	Himbert 2020	Singh 2020	Yang 2020	Codima 2021	Ma 2021	Lee 2021	Pu 2021	Gravier 2021	Heredia-Ciuro 2021	Machado 2021	Zhou 2021	De Oliveira Vacchi 2022	Xu 2022	%Y	%N
**Title**																																	
1		Y	N	Y	Y	Y	Y	Y	Y	Y	Y	Y	Y	Y	Y	N	Y	Y	Y	Y	Y	Y	Y	Y	Y	Y	Y	Y	Y	Y	Y	93	7
**Abstract**																																	
2 i		Y	N	Y	Y	Y	Y	Y	Y	Y	Y	Y	Y	Y	Y	N	Y	Y	Y	Y	Y	Y	Y	Y	Y	Y	Y	Y	Y	Y	Y	93	7
2 ii		Y	Y	Y	Y	Y	Y	Y	Y	Y	Y	Y	Y	Y	Y	Y	Y	Y	Y	Y	Y	Y	Y	Y	Y	Y	Y	Y	Y	Y	Y	100	0
2 iii		Y	Y	Y	Y	Y	Y	Y	Y	PY	Y	PY	Y	Y	Y	PY	PY	Y	N	Y	Y	Y	PY	Y	Y	Y	Y	Y	Y	Y	Y	80	3
2 iv		Y	PY	Y	N	Y	PY	Y	PY	PY	PY	Y	PY	Y	Y	Y	PY	Y	N	Y	Y	Y	Y	Y	Y	N	Y	Y	N	Y	Y	63	13
2 v		PY	N	N	N	N	N	N	N	Y	Y	N	N	N	Y	N	N	N	N	Y	N	Y	N	N	N	N	Y	Y	N	Y	N	27	70
2 vi		N	N	Y	Y	Y	Y	N	N	N	Y	N	Y	N	PY	PY	N	PY	N	Y	Y	N	N	Y	Y	N	N	Y	Y	N	Y	43	47
2 vii		PY	PY	PY	Y	Y	Y	PY	Y	Y	N	PY	Y	Y	Y	Y	Y	PY	N	Y	Y	Y	Y	Y	PY	Y	Y	Y	PY	PY	Y	63	7
2 viii		PY	N	Y	Y	Y	Y	Y	Y	Y	N	PY	PY	Y	Y	N	Y	Y	N	Y	PY	Y	Y	N	Y	Y	Y	Y	Y	Y	Y	70	17
2 ix		Y	PY	N	N	Y	PY	PY	N	Y	N	N	Y	Y	Y	N	N	N	N	N	N	N	N	N	N	Y	Y	N	N	N	N	27	63
2 x		Y	Y	Y	Y	Y	Y	Y	Y	Y	Y	Y	Y	Y	Y	Y	Y	PY	Y	Y	Y	Y	PY	PY	Y	Y	Y	Y	Y	Y	Y	90	0
2 xi		N	N	N	N	N	N	N	N	N	N	N	N	N	N	N	N	N	N	N	N	N	N	N	N	N	N	N	N	N	N	0	100
2 xii		N	N	N	N	N	Y	N	N	N	N	N	N	N	N	N	N	N	N	N	N	N	N	N	N	N	Y	N	N	Y	N	10	90
**Introduction**																																	
3		Y	Y	Y	Y	Y	Y	Y	Y	Y	Y	Y	Y	Y	Y	Y	Y	Y	Y	Y	Y	Y	Y	Y	Y	Y	Y	Y	Y	Y	Y	100	0
4		Y	Y	Y	Y	Y	Y	Y	Y	Y	Y	Y	Y	Y	Y	Y	Y	Y	Y	Y	Y	Y	Y	Y	Y	Y	Y	Y	Y	Y	Y	100	0
**Methods**																																	
5		Y	Y	Y	Y	Y	Y	Y	Y	Y	Y	Y	Y	Y	Y	Y	Y	Y	Y	Y	Y	Y	Y	Y	Y	Y	Y	Y	Y	Y	Y	100	0

6	Y	Y	Y	Y	Y	Y	Y	Y	Y	PY	Y	Y	Y	Y	Y	Y	Y	Y	Y	Y	Y	Y	Y	Y	Y	Y	Y	Y	Y	Y	97	0
7	Y	PY	PY	Y	Y	Y	PY	Y	PY	Y	N	Y	Y	Y	N	PY	PY	Y	Y	N	PY	Y	N	Y	PY	N	Y	N	Y	Y	53	20
8	Y	Y	N	Y	Y	Y	Y	Y	PY	Y	Y	Y	Y	Y	PY	PY	Y	Y	Y	Y	Y	Y	Y	Y	Y	Y	Y	Y	Y	Y	87	3
9	Y	Y	Y	Y	Y	Y	Y	Y	Y	Y	PY	Y	Y	Y	Y	PY	N	N	Y	Y	Y	Y	N	Y	Y	N	Y	Y	Y	Y	80	13
10a	Y	Y	Y	Y	Y	Y	PY	Y	Y	Y	Y	Y	Y	Y	Y	Y	Y	Y	Y	Y	Y	Y	PY	Y	Y	Y	Y	Y	Y	Y	93	0
10b	Y	Y	PY	Y	Y	PY	PY	Y	Y	N	Y	Y	Y	Y	Y	PY	Y	PY	Y	Y	Y	Y	N	Y	Y	N	Y	Y	Y	N	70	13
11	Y	Y	Y	Y	Y	Y	Y	Y	Y	Y	Y	Y	Y	Y	Y	PY	Y	Y	Y	Y	PY	Y	Y	Y	Y	PY	Y	Y	Y	Y	90	0
12	NA	NA	Y	Y	Y	Y	PY	Y	Y	Y	Y	Y	Y	Y	Y	Y	NA	NA	Y	Y	NA	Y	Y	N	Y	Y	Y	Y	Y	Y	92	4
13a	Y	N	Y	Y	Y	Y	PY	Y	Y	Y	Y	Y	Y	Y	Y	Y	Y	Y	Y	Y	Y	Y	Y	Y	Y	Y	Y	Y	Y	Y	93	3
13b	NA	NA	Y	Y	Y	Y	N	Y	Y	Y	PY	Y	Y	Y	N	PY	N	NA	Y	PY	NA	PY	PY	N	Y	Y	Y	Y	N	Y	62	19
13c	Y	N	Y	Y	Y	Y	Y	Y	Y	Y	Y	Y	Y	Y	Y	Y	Y	Y	Y	Y	Y	Y	N	Y	Y	Y	Y	Y	Y	Y	93	7
13d	Y	N	Y	Y	Y	Y	Y	Y	Y	Y	Y	Y	Y	Y	Y	Y	PY	Y	Y	Y	Y	Y	Y	Y	Y	Y	Y	Y	Y	Y	93	3
13e	NA	NA	Y	NA	NA	NA	NA	Y	Y	Y	Y	Y	Y	Y	Y	PY	NA	NA	Y	N	NA	Y	NA	N	Y	NA	Y	N	NA	Y	78	17
13f	NA	NA	Y	Y	Y	Y	NA	NA	Y	Y	Y	NA	Y	Y	Y	NA	NA	NA	Y	Y	NA	Y	NA	N	Y	NA	Y	Y	NA	NA	94	6
14	Y	PY	PY	Y	Y	PY	Y	PY	Y	Y	Y	Y	Y	Y	Y	Y	Y	Y	Y	Y	N	Y	PY	PY	Y	N	Y	PY	PY	Y	67	7
15	N	N	N	N	Y	Y	N	Y	Y	Y	N	N	Y	Y	N	Y	N	PY	N	N	N	N	N	Y	Y	N	N	N	Y	N	37	60
**Results**																																
16a	Y	Y	Y	Y	Y	Y	Y	Y	Y	Y	Y	Y	Y	Y	Y	Y	Y	Y	Y	Y	Y	Y	Y	Y	Y	Y	Y	Y	Y	Y	100	0
16b	Y	Y	N	N	Y	N	N	Y	N	Y	N	Y	Y	Y	N	Y	N	N	N	N	Y	N	N	N	N	N	N	N	N	N	33	67
17	Y	Y	Y	Y	Y	Y	Y	Y	Y	Y	Y	Y	Y	Y	Y	Y	Y	Y	Y	Y	Y	Y	Y	Y	Y	Y	Y	Y	Y	Y	100	0
18	Y	Y	N	Y	Y	Y	Y	Y	Y	Y	Y	Y	Y	Y	Y	N	Y	Y	Y	N	Y	Y	Y	Y	Y	Y	Y	Y	Y	Y	90	10
19	PY	PY	N	Y	Y	PY	Y	N	Y	Y	N	N	Y	Y	Y	Y	N	N	N	N	N	Y	Y	Y	Y	Y	PY	Y	Y	PY	53	30
20a	PY	PY	PY	PY	Y	PY	Y	PY	PY	Y	PY	PY	Y	Y	N	PY	Y	Y	PY	PY	N	PY	N	N	Y	N	N	N	Y	N	30	27
20b	NA	NA	Y	Y	Y	Y	Y	Y	Y	Y	Y	Y	Y	Y	PY	Y	NA	NA	Y	Y	NA	Y	Y	Y	Y	Y	Y	Y	Y	Y	96	0
20c	Y	NA	Y	NA	NA	NA	NA	Y	Y	Y	Y	PY	NA	Y	Y	N	NA	NA	Y	PY	NA	Y	NA	Y	Y	NA	Y	PY	NA	Y	78	6
20d	NA	NA	Y	Y	NA	Y	NA	NA	Y	PY	Y	NA	NA	Y	Y	NA	NA	NA	Y	N	NA	Y	NA	Y	Y	NA	Y	N	NA	NA	81	13
21	PY	PY	N	Y	Y	N	N	PY	PY	Y	PY	Y	Y	Y	PY	N	Y	Y	PY	Y	N	Y	PY	PY	Y	N	Y	PY	PY	Y	43	20

22	N	N	N	N	Y	Y	N	Y	Y	Y	N	N	Y	Y	N	Y	N	N	N	N	N	N	N	Y	Y	N	N	N	Y	N	37	63
**Discussion**																																
23a	Y	Y	Y	Y	Y	Y	Y	Y	Y	Y	Y	Y	Y	Y	Y	Y	Y	Y	Y	Y	Y	Y	Y	Y	Y	Y	Y	Y	Y	Y	100	0
23b	Y	Y	N	Y	Y	Y	N	Y	Y	Y	Y	Y	Y	Y	Y	Y	Y	Y	Y	Y	Y	Y	Y	Y	Y	Y	Y	Y	Y	Y	93	7
23c	Y	Y	Y	Y	Y	Y	Y	Y	Y	Y	Y	N	Y	Y	PY	N	Y	N	N	Y	N	N	N	N	N	PY	Y	Y	N	Y	60	33
23d	Y	Y	PY	Y	Y	Y	Y	Y	Y	Y	Y	Y	Y	Y	Y	PY	Y	PY	Y	Y	Y	Y	PY	PY	Y	Y	Y	Y	N	Y	80	3
**Other Information**																																
24a	N	N	N	N	N	Y	Y	Y	N	Y	N	N	N	N	N	Y	N	N	N	N	Y	Y	N	N	Y	Y	Y	N	Y	N	37	63
24b	Y	N	N	N	N	Y	N	N	N	Y	N	N	Y	Y	N	N	N	N	N	N	N	N	N	N	N	N	N	N	N	N	17	83
24c	Y	N	N	N	Y	N	N	N	N	Y	N	N	Y	Y	N	N	N	N	N	N	N	N	N	N	Y	N	N	N	N	N	20	80
25	Y	Y	Y	Y	Y	N	Y	Y	Y	N	N	Y	Y	Y	N	Y	N	Y	Y	Y	N	Y	N	N	Y	Y	Y	Y	N	Y	70	30
26	Y	Y	Y	Y	Y	Y	Y	Y	Y	Y	Y	Y	Y	Y	PY	Y	N	Y	Y	Y	Y	Y	Y	N	Y	Y	Y	Y	Y	Y	90	7
27	N	N	N	PY	N	N	N	N	N	N	N	N	N	N	N	N	N	N	N	N	N	N	N	N	PY	N	Y	N	N	N	3	90
**Scoring (total of 53)**																																
Y	34	22	31	38	44	38	29	38	39	42	31	36	45	48	28	28	26	25	39	33	29	37	24	32	43	33	43	33	35	37		
PY	6	8	6	2	0	6	7	4	6	3	7	4	0	1	7	11	5	3	2	4	2	4	6	4	2	2	1	4	3	1		
N	7	16	16	13	6	7	13	9	8	8	15	11	6	4	18	12	16	18	12	16	15	12	19	17	8	14	9	16	11	13		
NA	6	7	0	4	3	2	4	2	0	0	0	2	2	0	0	2	6	7	0	0	7	0	4	0	0	4	0	0	4	2		

Footnote: PRISMA 2020 ratings: Y=yes, N=no, PY=partial yes, NA=not applicable

Table S8. Exercise interventions from RCTs included in meta-analyses for overview primary outcomes

Systematic review (author)	Outcome	Type of exercise intervention and number of RCTs
**Pre-treatment reviews**		
**Exercise Capacity**		
Cavalheri 2017[68]	6MWT (m)	Aerobic + respiratory x1,<br>Aerobic + respiratory (incl. IMT) + balance + stretching x1
Rosero 2019[69]	6MWT	Respiratory (incl IMT) x2,<br>Aerobic + respiratory (incl. IMT) x1,<br>Aerobic + resistance x1,<br>Aerobic + resistance + respiratory + stretching x1, Aerobic + resistance + respiratory x1
Li 2019[70]	6MWT (m)	Aerobic + respiratory (incl IMTx1) x2,<br>Aerobic + resistance + respiratory + stretching x1,<br>Aerobic + resistance x1,
Pu 2021[71]	6MWT (m)	Aerobic + respiratory x3,<br>Respiratory x1
Gravier 2021[72]	6MWT (m)	Aerobic + respiratory x2<br>Aerobic + resistance + respiratory (incl IMT) + balance + stretching x1,<br>1x AT+BE,<br>Aerobic + resistance x1,<br>Aerobic + resistance + respiratory x1
de Oliveira Vacchi 2022[73]	6MWT (m)	Aerobic + respiratory (IMT) x3,<br>Aerobic + resistance + respiratory (IMT) x1
Rosero 2019[69]	$VO_{2\ peak}$	Aerobic + respiratory (IMT) x 2<br>Aerobic + resistance x1
Li 2019[70]	$VO_{2\ peak}$ (mL/kg/min)	Aerobic + resistance x1,<br>Aerobic + respiratory x1
Gravier 2021[72]	$VO_{2\ peak}$ (mL/kg/min)	Aerobic + resistance x1,<br>Aerobic + respiratory x1

**Health-related quality of life**		
Rosero 2019[69]	Questionnaire (details NR)	Respiratory (IMT) x1,<br>Aerobic + respiratory (incl IMT x1) x2,<br>Aerobic + resistance + respiratory + stretching x1,
Pu 2021[71]	EORTC QLQ-C3030	Aerobic + respiratory x3
Gravier 2021[72]	EORTC QLQ-C3030	Aerobic + respiratory x2
**Pulmonary complications**		
Cavalheri 2017[68]	PPC	Aerobic + respiratory x2<br>Aerobic + resistance + respiratory (incl IMT) x1<br>Aerobic + respiratory (IMT) + balance + stretching x1
Rosero 2019[69]	PPC	Aerobic and respiratory (incl IMT) x3,<br>Aerobic + respiratory (incl IMT) + stretching x1,<br>Respiratory (IMT) x1,<br>Aerobic x1,<br>Aerobic + resistance x1<br>Respiratory (incl IMT) x1
Li 2019[70]	PPC	Aerobic + respiratory (incl IMTx1) x2<br>Aerobic + resistance + respiratory x1<br>Aerobic + respiratory (incl IMT) + stretching x1,<br>Aerobic + resistance x1<br>Respiratory x1
Pu 2021[71]	PPC	Aerobic + respiratory x3<br>Aerobic + resistance + respiratory (incl IMT) x1<br>Respiratory x1
de Oliveira Vacchi 2022[73]	Post-operative complication (pnuemonia)	Aerobic + respiratory (IMT) x3<br>Respiratory (incl IMT) x1<br>Aerobic + resistance + respiratory (incl IMT) x1
de Oliveira Vacchi 2022[73]	Post-operative complication (atelectasis)	Aerobic + respiratory (IMT) x3<br>Aerobic + resistance + respiratory (incl IMT) x1

de Oliveira Vacchi 2022[73]	Post-operative complication (MV>48 hrs post-op)	Aerobic + respiratory (IMT) x2<br>Aerobic + resistance + respiratory (IMT) x1
**Post-treatment reviews**		
**Exercise capacity**		
Li 2017[74]	6MWT (m)	Aerobic + resistance x4
Sommer 2018[75]	Exercise Capacity (VO_2 peak or 6MWT) – 12-20 wk f/u	Aerobic + resistance x2<br>Aerobic + resistance + whole body vibration x1<br>Aerobic + resistance + respiratory (IMT) x1
	Exercise Capacity (VO_2 peak or 6MWT) - long term follow-up (1 year)	Aerobic + resistance x1
	Exercise Capacity - early initiated intervention	Aerobic + resistance x1
	Exercise Capacity - late initiated intervention	Aerobic + resistance x2<br>Aerobic + resistance + whole body vibration x1<br>Aerobic + resistance + respiratory (IMT) x1
Cavalheri 2019[76]	6MWT (m)	Aerobic + resistance x4<br>Aerobic + resistance + whole body vibration x1
	$VO_{2\,peak}$ (mL/kg/min)	Aerobic + resistance + whole body vibration x1<br>Aerobic + respiratory (IMT) x1<br>Aerobic + resistance x1<br>Aerobic + resistance + respiratory (IMT) x1
**Physical function**		
Cavalheri 2019[76]	Force-generating capacity (quadriceps)	Aerobic + respiratory (IMT) x1<br>Aerobic + resistance x2<br>Aerobic + resistance + whole body vibration x1
	Force-generating capacity (grip strength) kg	Aerobic + resistance + respiratory (IMT) x1<br>Aerobic + resistance x1
**Health-related quality of life**		

Li 2017[74]	Physical (SF-36)	Aerobic + resistance x2<br>Aerobic + resistance + respiratory (IMT) x1
	Mental (SF-36)	Aerobic + resistance x1<br>Aerobic + resistance + respiratory (IMT) x1
Sommer 2018[75]	Physical - short term (12-20 weeks) - (EORTC QLQ-C30 + SF-36)	Aerobic + resistance x2<br>Aerobic + resistance + respiratory (IMT) x1<br>Aerobic + resistance + whole body vibration x1
	Physical - long term (1 year) (EORTC QLQ-C3030 + SF-36)	Aerobic + resistance x1
	Mental - short term (12-20 weeks) (EORTC QLQ-C30 + SF-36)	NR
	Mental - long term (1 year) - (EORTC QLQ-C30 + SF-36) -	Aerobic + resistance x1
Cavalheri 2019[76]	Physical (SF-36)	Aerobic + resistance x3<br>Aerobic + resistance + respiratory (IMT) x1
	Mental (SF-36)	Aerobic + resistance x3<br>Aerobic + resistance + respiratory (IMT) x1
	Global (EORTC-QLQ-C30)	Aerobic + resistance x2<br>Aerobic + resistance + whole body vibration x1<br>Aerobic + respiratory (IMT) x1
	Functional (EORTC-QLQ-C30)	Aerobic + resistance x2
	Symptoms (EORTC-QLQ-C30)	Aerobic + resistance x2
	Physical functioning (EORTC-QLQ-C30)	Aerobic + resistance + whole body vibration x1<br>Aerobic + respiratory (IMT) x1
**Pulmonary complications**		
Li 2017[74]	PPCs	Aerobic + resistance x3

Larsen 2019[77]	PPCs	Aerobic + resistance x2
**Pre and post-treatment reviews**		
**Exercise capacity**		
Wang 2018[78]	6MWT (m)	Respiratory + aerobic x2<br>Respiratory + aerobic + resistance x1<br>Respiratory + aerobic + resistance +stretching x1<br>Respiratory (IMT) x1
**Health-related quality of life**		
Machado 2021[79]	Physical (SF-36, EORTC-QLQ-C30, FACT-L)	Aerobic (x1 including HIIT) + resistance x2,<br>Aerobic +balance x1,<br>Aerobic (including HIIT) + resistance + respiratory + stretching x1,<br>Aerobic (x1 including HIIT) + resistance + respiratory (IMT) x2,<br>Aerobic (including HIIT) + resistance + respiratory x1
	Mental (SF-36)	Aerobic (x1 including HIIT) + resistance x2,<br>Aerobic (including HIIT) + resistance + respiratory (x1 including IMT) x2,<br>Aerobic +balance x1,
	Emotional (EORTC-QLQ-C30, FACT-L)	Aerobic (including HIIT) + resistance x1,<br>Aerobic (including HIIT) + resistance + respiratory + stretching x1,<br>Aerobic + resistance + respiratory (IMT) x1
	Global (EORTC-QLQ-C30, FACT-L, Saint-George Respiratory Questionnaire	Aerobic (x1 including HIIT) + resistance x3,<br>Aerobic (including HIIT) + resistance + respiratory + stretching x1,<br>Aerobic + resistance + respiratory (IMT) x1
**Pulmonary complications**		
Wang 2018[78]	PPCs	Respiratory x4 (1 study included IMT)<br>Respiratory + aerobic x2<br>Respiratory (1 study included IMT)) + aerobic +resistance x2
	Subgroup: respiratory only	Respiratory (1 study included IMT)
	Subgroup: respiratory plus aerobic/resistance	Respiratory + aerobic x2<br>Respiratory (1 study included IMT)) + aerobic +resistance x2

Xu 2022[80]	PPCs (pre-op studies)	Aerobic + resistance + respiratory (including IMT) x2,<br>Aerobic (1 including HIIT) + resistance + respiratory x2,<br>Aerobic + respiratory (1 including IMT) x5,<br>Aerobic (HIIT) + resistance x1,
	PPCs (post-op studies)	Respiratory x3,<br>Aerobic + resistance x1,<br>Aerobic + respiratory + ROM x1
**Pre, during or post-treatment reviews**		
**Exercise capacity**		
Liu 2019[81]	6MWT (m)	Respiratory x6 (x2 incl IMT)<br>Respiratory + aerobic + resistance x3
	Subgroup:<br>Breathing exercise only	Respiratory x6 (x2 including IMT)
	Subgroup: Breathing and other exercise	Respiratory + aerobic + resistance x3
Zhou 2021[82]	6MWT (m)	Aerobic and resistance x3<br>Aerobic x3
Heredia-Ciuro 2021[83]	$VO_{2\ peak}$ (mL/kg/min)	HIIT x3,<br>HIIT + resistance + respiratory (IMT) x2,<br>HIIT + resistance x1
Singh 2020[84]	Aerobic Fitness	NR
	Subgroup – Aerobic ex	NR
	Subgroup -Combined ex	NR
	Subgroup – Supervised	NR
	Subgroup -<br>Unsupervised	NR
	Subgroup - <12/52	NR

	Subgroup - ≥12/52	NR
**Physical function**		
Singh 2020[84]	UL Strength	NR
	Subgroup – Aerobic ex	NR
	Subgroup – Combined ex	NR
	Subgroup - <12/52	NR
	Subgroup - ≥12/52	NR
	LL Strength	NR
	Subgroup – Aerobic ex	NR
	Subgroup – Combined ex	NR
	Subgroup - Supervised	NR
	Subgroup - Unsupervised	NR
	Subgroup - <12/52	NR
	Subgroup - ≥12/52	NR
**Health-related quality of life**		
Singh 2020[84]	Global (EORTC-QLQ-C30, FACT-L, SF36, WHO QoL Bref)	NR
	Subgroup – Aerobic ex	NR
	Subgroup - Combined	NR

	Subgroup - Supervised	NR
	Subgroup - Unsupervised	NR
	Subgroup - <12/52	NR
	Subgroup - ≥12/52	NR
Zhou 2021[82]	Global (FACT-L, EORTC-QLQ-C30, SF36)	Aerobic x3,<br>Aerobic + resistance x3,<br>Aerobic + balance x1
**During and/or post treatment**		
**Exercise capacity**		
Peddle-McIntyre 2019[85]	6MWT (meters)	Aerobic, resistance and respiratory x2<br>Aerobic and resistance x1
Lee 2021[86]	6MWT (meters)	Aerobic, resistance and respiratory x2<br>Aerobic x2
**Physical function**		
Lee 2021[86]	UL Strength	Aerobic, resistance and respiratory x2
**Health-related quality of life**		
Peddle-McIntyre 2019[85]	Global (EORTC-QLQ-C30, Chronic Respiratory Disease Questionnaire)	Aerobic, resistance and respiratory x1<br>Aerobic x1<br>Respiratory (IMT) x1
	Physical (EORTC-QLQ-C30 and SF36)	Aerobic, resistance and respiratory x2<br>Aerobic x1
Lee 2021[86]	Physical (FACT-L)	Aerobic x2
	Social (FACT-L)	Aerobic x2
	Emotional (FACT-L)	Aerobic x2

	Functional (FACT-L)	Aerobic x2
	General (FACT-L)	Aerobic, resistance and respiratory x1<br>Aerobic x1
	Total score (FACT-L)	Aerobic x2

Figure S1. Correlation correction matrix

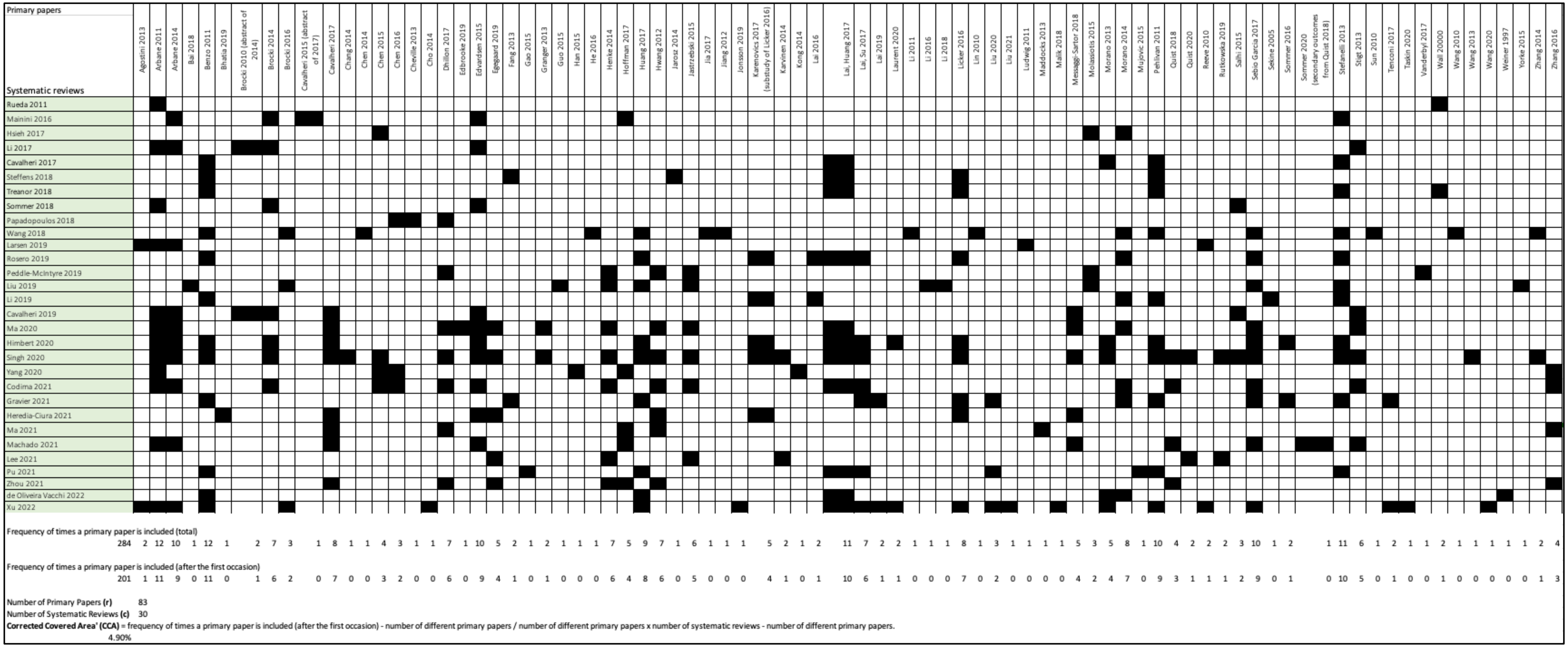

Footnote: rows indicate each systematic review included in the overview and columns indicate the RCTs included within each systematic review. The formula used to calculate the corrected covered area (CCA) was $\text{CCA} = \frac{N-r}{r\,c-r}$, where r= the number of included primary studies, c= the number of included SRs and N= the frequency a primary paper is included after the first occasion.

Figure S2. Pre, during and/or post treatment (surgical and non-surgical) – meta-analysis findings for overview primary outcomes

1) Exercise capacity

a) 6MWT (MD)

Systematic review	RCTs	n	MD (95% CI)	I^2	GRADE	AMSTAR-2
**Liu et al 2019**	9	557	37.72 (15.06 to 60.37)	71%	Low	Critically low
Subgroup: respiratory only	6	489	35.66 (8.12 to 63.2)	94%		Critically low
Subgroup: respiratory + other	3	68	41.2 (2.49 to 79.92)	0%		Critically low
**Zhou et al 2021**	5	383	20.4 (-14.15 to 54.95)	44%	Very low	Critically low

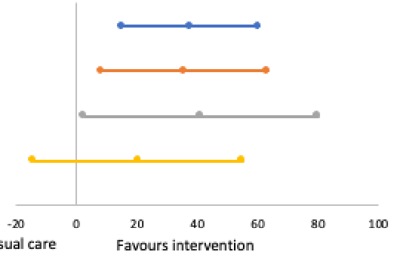

b) 6MWT and $VO_{2\ peak}$ (SMD)

Systematic review	RCTs	n	SMD (95% CI)	I^2	GRADE	AMSTAR-2
**Singh et al 2020**	26	1778	0.54 (0.32 to 0.76)	78%	NR	Critically low
Subgroup: Aerobic only	11	827	0.44 (0.06 to 0.82)	85%	NR	Critically low
Subgroup: Combined	15	951	0.6 (0.35 to 0.86)	68%	NR	Critically low
Subgroup: Supervised	23	1634	0.54 (0.32 to 0.76)	77%	NR	Critically low
Subgroup: Unsupervised	3	144	0.95 (-0.25 to 2.16)	88%	NR	Critically low
Subgroup: < 12 weeks	19	1075	0.61 (0.29 to 0.93)	82%	NR	Critically low
Subgroup: ≥ 12 weeks	7	703	0.42 (0.27 to 0.57)	1%	NR	Critically low

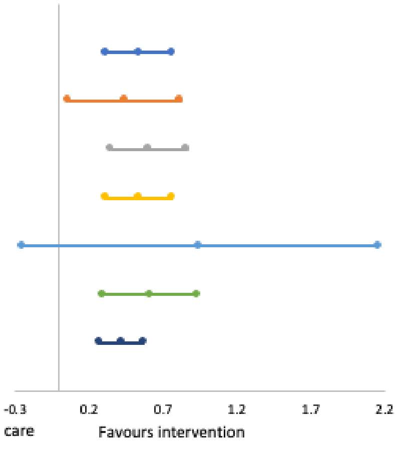

c) $VO_{2\ peak}$ (MD)

Systematic review	RCTs	n	SMD (95% CI)	I^2	GRADE	AMSTAR-2
**Heredia-Ciuro 2021**	6	286	2.62 (1.55 to 3.68)	0%	Moderate	Critically low

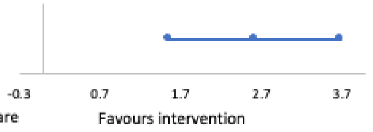

2) Physical function (SMD)

Systematic review	RCTs	n	SMD (95% CI)	I^2	GRADE	AMSTAR-2
**Singh 2020 - Upper limb strength**	9	721	0.59 (0.30 to 0.88)	66%	Low	Critically low
Subgroup: Aerobic only	1	111	0.14 (-0.23 to 0.51)			Critically low
Subgroup: Combined	8	610	0.69 (0.36 to 1.01)	68%		Critically low
Subgroup: < 12 weeks	6	231	0.90 (0.32 to 1.48)	71%		Critically low
Subgroup: ≥ 12 weeks	3	490	0.36 (0.16 to 0.57)	0%		Critically low
**Singh 2020 - Lower limb strength**	10	660	0.38 (0.16 to 0.61)	48%	Low	Critically low
Subgroup: Aerobic only	2	134	0.32 (-0.03 to 0.66)	0%		Critically low
Subgroup: Combined	8	526	0.42 (0.14 to 0.70)	59%		Critically low
Subgroup: Supervised	8	531	0.42 (0.25 to 0.59)	6%		Critically low
Subgroup: Unsupervised	2	129	0.28 (-0.72 to 1.28)	87%		Critically low
Subgroup: < 12 weeks	6	281	0.28 (-0.10 to 0.66)	52%		Critically low
Subgroup: ≥ 12 weeks	4	379	0.47 (0.27 to 0.68)	8%		Critically low

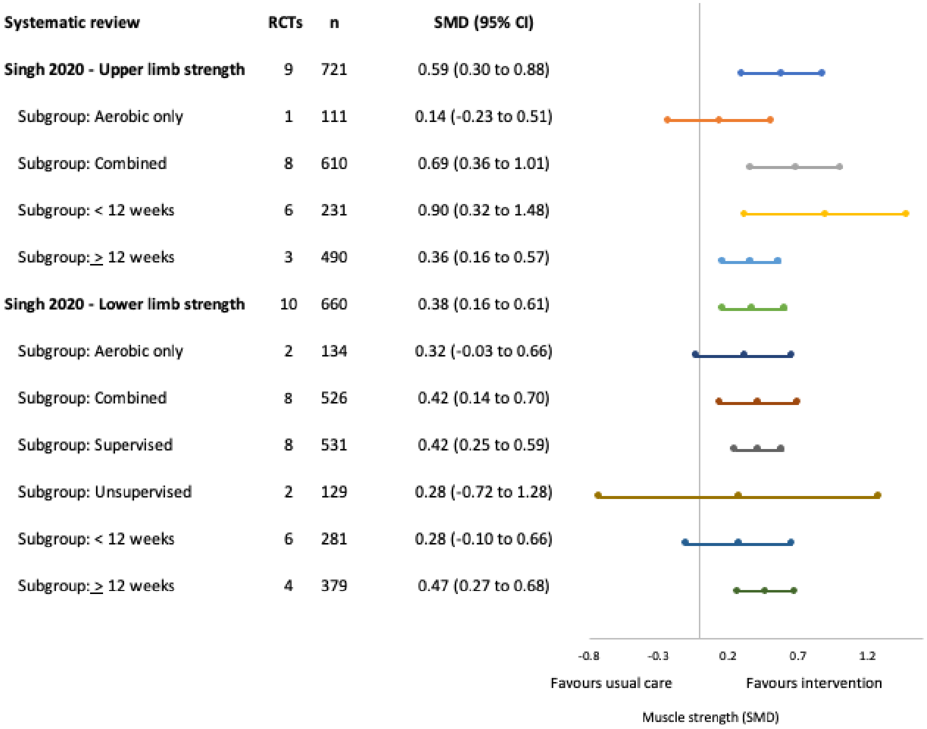

3) HRQoL (SMD)

Systematic review	RCTs	n	SMD (95% CI)	I^2	GRADE	AMSTAR-2
**Global**						
**Singh 2020**	20	1293	0.31 (0.20 to 0.42)	0%	NR	Critically low
Subgroup: Aerobic only	9	492	0.31 (0.14 to 0.49)	0%	NR	Critically low
Subgroup: Combined	11	801	0.31 (0.15 to 0.47)	13%	NR	Critically low
Subgroup: Supervised	17	1139	0.36 (0.24 to 0.48)	0%	NR	Critically low
Subgroup: Unsupervised	3	154	-0.02 (-0.33 to 0.30)	0%	NR	Critically low
Subgroup: < 12 weeks	15	700	0.27 (0.12 to 0.42)	0%	NR	Critically low
Subgroup: ≥ 12 weeks	5	593	0.35 (0.13 to 0.57)	47%	NR	Critically low
**Zhou 2021**	7	479	0.33 (0.08 to 0.58)	25%	Low	Critically low

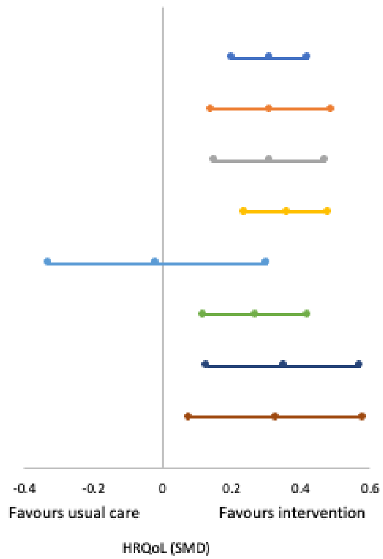

Figure S3. During and/or post treatment (non-surgical) – meta-analysis findings for overview primary outcomes

1) Exercise capacity
   a) 6MWT (MD)

Systematic review	RCTs	n	MD (95% CI)	I^2	GRADE	AMSTAR-2
**Peddle-McIntyre 2019**	3	59	63.33 (3.7 to 122.96)	0%	Low	Low

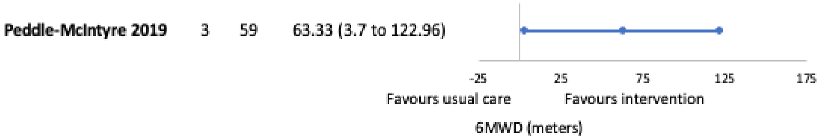

2) 6MWT (SMD)

Systematic review	RCTs	n	SMD (95% CI)	I^2	GRADE	AMSTAR-2
**Lee 2021**	**4**	290	0.38 (-0.42 to 1.18)	80%	Very low	Critically low

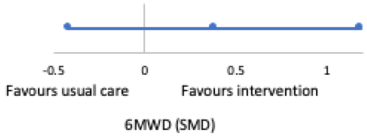

3) Physical function (SMD)

Systematic review	RCTs	n	SMD (95% CI)	I^2	GRADE	AMSTAR-2
**Lee 2021 - Upper limb strength**	2	59	1.39 (0.80 to 1.98)	0%	Low	Critically low

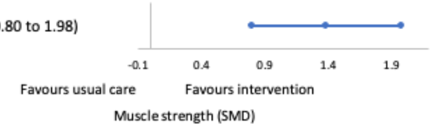

4) HRQoL (SMD)

Systematic review	RCTs	n	SMD (95% CI)	I^2	GRADE	AMSTAR-2
**Global**						
Peddle-McIntyre 2019	3	90	0.51 (0.08 to 0.93)	0%	Low	Low
**Physical**						
Peddle-McIntyre 2019	3	73	0.11 (-0.36 to 0.58)	0%	Low	Low
Lee 2021	2	231	0.46 (0.19 to 0.72)	31%	Moderate	Critically low
**Social**						
Lee 2021	2	231	0.39 (0.13 to 0.66)	5%	Moderate	Critically low
**Emotional**						
Lee 2021	2	231	0.07 (-0.19 to 0.33)	0%	Low	Critically low
**Functional**						
Lee 2021	2	231	0.30 (0.05 to 0.56)	0%	Moderate	Critically low
**General**						
Lee 2021	2	231	0.39 (0.13 to 0.65)	17%	Moderate	Critically low
**Total score**						
Lee 2021	2	231	0.15 (-0.71 to 1.00)	59%	Moderate	Critically low

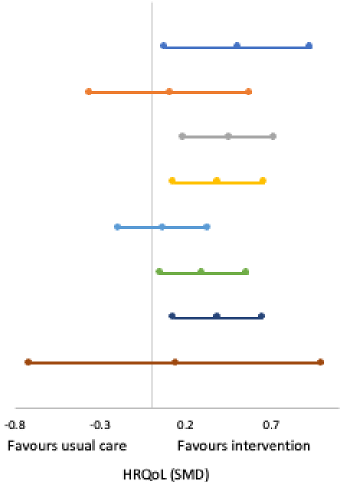

References

1. Archer, K. and H. Ciechanowicz, *The effectiveness of preoperative pulmonary rehabilitation in reducing postoperative pulmonary complications in lung cancer: a systematic review and meta-analysis...The Chartered Society of Physiotherapy UK Conference 2018, Birmingham, UK, 19-20 October 2018.* Physiotherapy, 2019. **105**: p. e211-e212 DOI: 10.1016/j.physio.2018.11.233.
2. Barakat, S., et al., *Does Health Coaching Grow Capacity in Cancer Survivors? A Systematic Review.* Population Health Management, 2018. **21**(1): p. 63-81 DOI: http://dx.doi.org/10.1089/pop.2017.0040.
3. Bayly, J., et al., *Changing health behaviour with rehabilitation in thoracic cancer: A systematic review and synthesis.* Psycho-Oncology, 2018. **27**(7): p. 1675-1694 DOI: http://dx.doi.org/10.1002/pon.4684.
4. Bibo, L., J. Goldblatt, and C. Merry, *Does preoperative pulmonary rehabilitation/physiotherapy improve patient outcomes following lung resection?* Interactive cardiovascular and thoracic surgery, 2021. **32**(6): p. 933-937 DOI: https://dx.doi.org/10.1093/icvts/ivab011.
5. Castro, G., et al., *The ideal amount of physical activity to improve quality of life and symptoms in lung cancer patients: A systematic review.* Journal of Clinical Oncology, 2019. **37**(Supplement 15) DOI: http://dx.doi.org/10.1200/JCO.2019.37.15_suppl.e23158.
6. Chan, C.L.W., et al., *A systematic review of the effectiveness of qigong exercise in supportive cancer care.* Supportive Care in Cancer, 2012. **20**(6): p. 1121-1133 DOI: http://dx.doi.org/10.1007/s00520-011-1378-3.
7. Clark, E., et al., *The effects of physical activity, fast-mimicking diet and psychological interventions on cancer survival: A systematic review and meta-analysis of randomized controlled trials.* Complementary Therapies in Medicine, 2021. **57**: p. 102654 DOI: http://dx.doi.org/10.1016/j.ctim.2020.102654.
8. Crandall, K., et al., *Exercise intervention for patients surgically treated for Non-Small Cell Lung Cancer (NSCLC): A systematic review.* Surgical Oncology, 2014. **23**(1): p. 17-30 DOI: http://dx.doi.org/10.1016/j.suronc.2014.01.001.
9. Curry, J., et al., *Feasibility, acceptability, and efficacy of online supportive care for individuals living with and beyond lung cancer: a systematic review.* Supportive Care in Cancer, 2021. **29**(11): p. 6995-7011 DOI: http://dx.doi.org/10.1007/s00520-021-06274-x.
10. Devor, S.T., et al., *Resistance exercise interventions during and following cancer treatment: A systematic review.* Journal of Supportive Oncology, 2013. **11**(2): p. 45-60 DOI: http://dx.doi.org/10.12788/j.suponc.0002.
11. Dittus, K.L., P.A. Ades, and R.E. Gramling, *Exercise interventions for individuals with advanced cancer: A systematic review.* Preventive Medicine, 2017. **104**: p. 124-132 DOI: http://dx.doi.org/10.1016/j.ypmed.2017.07.015.
12. dos Santos Goes, J. and G. Santos do Nascimento, *The degree of satisfaction of patients with cancer interned in a specialized oncology sector in relation to the assistance provided by the physiotherapy professional: integrative review.* Saude Coletiva, 2021. **11**(71): p. 9239-9245.
13. Driessen, E.J., et al., *Effects of prehabilitation and rehabilitation including a home-based component on physical fitness, adherence, treatment tolerance, and recovery in patients with non-small cell lung cancer: a systematic review.* Critical Reviews in Oncology/Hematology 2017 Jun;114:63-76, 2017.
14. Du, S., et al., *Patient education programs for cancer-related fatigue: A systematic review.* Patient Education and Counseling, 2015. **98**(11): p. 1308-1319 DOI: http://dx.doi.org/10.1016/j.pec.2015.05.003.
15. Dy, S.M., et al., *Interventions for Breathlessness in Patients With Advanced Cancer.* 2020.
16. Easwaran, K., et al., *Effectiveness of Tai Chi for health promotion for adults with health conditions: a scoping review of Meta-analyses.* Disability & Rehabilitation, 2021. **43**(21): p. 2978-2989 DOI: 10.1080/09638288.2020.1725916.

17. Ester, M., et al., *Current Evidence and Directions for Future Research in eHealth Physical Activity Interventions for Adults Affected by Cancer: Systematic Review.* JMIR Cancer, 2021. **7**(3): p. e28852 DOI: https://dx.doi.org/10.2196/28852.
18. Faithfull, S., et al., *Prehabilitation for adults diagnosed with cancer: A systematic review of long-term physical function, nutrition and patient-reported outcomes.* Eur J Cancer Care (Engl), 2019. **28**(4): p. e13023 DOI: 10.1111/ecc.13023.
19. Ferreira, V., et al., *Effects of preoperative nutrition and multimodal prehabilitation on functional capacity and postoperative complications in surgical lung cancer patients: a systematic review.* Supportive care in cancer : official journal of the Multinational Association of Supportive Care in Cancer, 2021 DOI: https://dx.doi.org/10.1007/s00520-021-06161-5.
20. García, R.S. and M.I. Yáñez Brage, *Effect of pre-operative pulmonary rehabilitation in lung cancer patients.* Rehabilitacion, 2013. **47**(4): p. 229-237 DOI: 10.1016/j.rh.2013.04.004.
21. Grande, A.J., et al., *Exercise for cancer cachexia in adults.* Cochrane Database of Systematic Reviews, 2021(3) DOI: 10.1002/14651858.CD010804.pub3.
22. Granger, C.L., et al., *Exercise intervention to improve exercise capacity and health related quality of life for patients with Non-small cell lung cancer: A systematic review.* Lung Cancer, 2011. **72**(2): p. 139-153 DOI: http://dx.doi.org/10.1016/j.lungcan.2011.01.006.
23. Groll, D.L., et al., *Fatigue and physical activity in older adults with cancer: A systematic review of the literature.* Cancer Nursing, 2007. **30**(5): p. E35-E45 DOI: http://dx.doi.org/10.1097/01.NCC.0000290815.99323.75.
24. Henshall, C.L., L. Allin, and H. Aveyard, *A Systematic Review and Narrative Synthesis to Explore the Effectiveness of Exercise-Based Interventions in Improving Fatigue, Dyspnea, and Depression in Lung Cancer Survivors.* Cancer Nursing, 2019. **42**(4): p. 295-306 DOI: 10.1097/NCC.0000000000000605.
25. Heywood, R., et al., *Safety and feasibility of exercise interventions in patients with advanced cancer: a systematic review.* Supportive Care in Cancer, 2017. **25**(10): p. 3031-3050 DOI: 10.1007/s00520-017-3827-0.
26. Heywood, R., T.L. Skinner, and A.L. McCarthy, *Efficacy of Exercise Interventions in Patients With Advanced Cancer: A Systematic Review.* Archives of Physical Medicine and Rehabilitation, 2018. **99**(12): p. 2595-2620 DOI: http://dx.doi.org/10.1016/j.apmr.2018.04.008.
27. Huang, C.H., et al., *Perioperative exercise intervention in patients with lung cancer: a systematic literature review of randomized controlled trials.* Ci Ji Yi Xue Za Zhi [Tzu-Chi Medical Journal] 2021 Oct-Dec;33(4):412-418, 2021.
28. Johnson, M.J. and D.C. Currow, *Treating breathlessness in lung cancer patients: the potential of breathing training.* Expert review of respiratory medicine, 2016. **10**(3): p. 241-3 DOI: https://dx.doi.org/10.1586/17476348.2016.1146596.
29. Kalter, J., et al., *Effects and moderators of exercise on quality of life and physical function in patients with cancer: An individual patient data meta-analysis of 34 RCTs.* Cancer Treatment Reviews, 2017. **52**: p. 91-104 DOI: http://dx.doi.org/10.1016/j.ctrv.2016.11.010.
30. Kasparov, B., et al., *Physical methods of rehabilitation (PMR) in patients with lung cancer.* Journal of clinical oncology, 2019. **37** DOI: 10.1200/JCO.2019.37.15_suppl.e23189.
31. Koeppel, M., et al., *Muscle hypertrophy in cancer patients and survivors via strength training. A meta-analysis and meta-regression.* Critical Reviews in Oncology/Hematology, 2021. **163**: p. 103371 DOI: http://dx.doi.org/10.1016/j.critrevonc.2021.103371.
32. Kuo, C.C., et al., *Clinical Effects of Baduanjin Qigong Exercise on Cancer Patients: A Systematic Review and Meta-Analysis on Randomized Controlled Trials.* Evidence-based Complementary and Alternative Medicine, 2021. **2021**: p. 6651238 DOI: http://dx.doi.org/10.1155/2021/6651238.
33. Li, R., et al., *The effect of the enhanced recovery after surgery program on lung cancer surgery: a systematic review and meta-analysis.* Journal of Thoracic Disease 2021 Jun;13(6):3566-3586, 2021.

34. Liu, W., et al., *Breathing exercises improve post-operative pulmonary function and quality of life in patients with lung cancer: a meta-analysis.* Experimental and Therapeutic Medicine 2013 Apr;5(4):1194-1200, 2013.
35. Lonbro, S., *The effect of progressive resistance training on lean body mass in post-treatment cancer patients - A systematic review.* Radiotherapy and Oncology, 2014. **110**(1): p. 71-80 DOI: http://dx.doi.org/10.1016/j.radonc.2013.07.008.
36. Lowe, S.S., S.M. Watanabe, and K.S. Courneya, *Physical activity as a supportive care intervention in palliative cancer patients: A systematic review.* Journal of Supportive Oncology, 2009. **7**(1): p. 27-34.
37. Ma, H.-L., et al., *Current evidence on traditional Chinese exercises for cancer-related fatigue: a quantitative synthesis of randomized controlled trials.* European Journal of Integrative Medicine, 2016. **8**(5): p. 707-714 DOI: http://dx.doi.org/10.1016/j.eujim.2016.05.007.
38. Makwana, N., A. Makwana, and N. Shetye, *Effect of exercise training on subjective and objective outcome in lung cancer.* Indian Journal of Physiotherapy and Occupational Therapy 2016 Apr-Jun;10(2):14-19, 2016.
39. Mao, X., et al., *The Clinical Value of Pulmonary Rehabilitation in Reducing Postoperative Complications and Mortality of Lung Cancer Resection: A Systematic Review and Meta-Analysis.* Frontiers in surgery, 2021. **8**: p. 685485 DOI: https://dx.doi.org/10.3389/fsurg.2021.685485.
40. Marthick, M., et al., *Supportive care interventions for people with cancer assisted by digital technology: Systematic review.* Journal of Medical Internet Research, 2021. **23**(10): p. e24722 DOI: https://dx.doi.org/10.2196/24722.
41. Medysky, M.E., et al., *Attention to the Principles of Exercise Training in Exercise Studies of Persons With Lung Cancer: A Systematic Review.* Journal of aging and physical activity, 2021: p. 1-11 DOI: http://dx.doi.org/10.1123/japa.2020-0269.
42. Meneses-Echavez, J.F., et al., *Prehabilitation Programs For Cancer Patients: A Systematic Review Of Randomized-controlled Trials...2021 ACSM Annual Meeting & World Congresses [Virtual], June 1 -5, 2021.* Medicine & Science in Sports & Exercise, 2021. **53**: p. 471-471 DOI: 10.1249/01.mss.0000764756.57953.e7.
43. Michael, C.M., et al., *Prehabilitation exercise therapy for cancer: A systematic review and meta-analysis.* Cancer Medicine, 2021. **10**(13): p. 4195-4205 DOI: http://dx.doi.org/10.1002/cam4.4021.
44. Nan, W., et al., *The Impact of Preoperative Exercise Therapy on the Surgical Outcomes of Patients with Lung Cancer and COPD: A Systematic Review and Meta-Analysis.* Journal of Thoracic Oncology, 2018. **13**(10 Supplement): p. S1015 DOI: http://dx.doi.org/10.1016/j.jtho.2018.08.1941.
45. Ni, H.-J., et al., *Exercise Training for Patients Pre- and Postsurgically Treated for Non-Small Cell Lung Cancer: A Systematic Review and Meta-analysis.* Integrative Cancer Therapies, 2017. **16**(1): p. 63-73 DOI: 10.1177/1534735416645180.
46. Paramanandam, V.S. and V. Dunn, *Role of exercise for the management of cancer-related fatigue in lung cancer: Systematic review.* Lung Cancer, 2013. **80**(SUPPL. 1): p. S52-S53.
47. Paramanandam, V.S. and V. Dunn, *Exercise for the management of cancer-related fatigue in lung cancer: a systematic review.* European Journal of Cancer Care, 2015. **24**(1): p. 4-14 DOI: 10.1111/ecc.12198.
48. Piraux, E., G. Caty, and G. Reychler, *Effects of preoperative combined aerobic and resistance exercise training in cancer patients undergoing tumour resection surgery: A systematic review of randomised trials.* Surgical Oncology, 2018. **27**(3): p. 584-594 DOI: http://dx.doi.org/10.1016/j.suronc.2018.07.007.
49. Pouwels, S., et al., *Preoperative exercise therapy in lung surgery patients: A systematic review.* Respiratory medicine, 2015. **109**(12): p. 1495-504 DOI: https://dx.doi.org/10.1016/j.rmed.2015.08.009.

50. Rathore, F.A. and A. Afridi, *Is exercise training effective within 12 months of lung resection for non-small cell lung cancer?-A Cochrane Review summary with commentary.* PM and R, 2021. **13**(3): p. 336-338 DOI: http://dx.doi.org/10.1002/pmrj.12564.
51. Rowntree, R.A. and H. Hosseinzadeh, *Lung Cancer and Self-Management Interventions: A Systematic Review of Randomised Controlled Trials.* International Journal of Environmental Research and Public Health, 2022. **19**(1): p. 536 DOI: https://dx.doi.org/10.3390/ijerph19010536.
52. Ream, E., et al., *Telephone interventions for symptom management in adults with cancer.* The Cochrane database of systematic reviews, 2020. **6**: p. CD007568 DOI: https://dx.doi.org/10.1002/14651858.CD007568.pub2.
53. Rodriguez-Larrad, A., et al., *Perioperative physiotherapy in patients undergoing lung cancer resection.* Interactive Cardiovascular and Thoracic Surgery 2014 Aug;19(2):269-281, 2014.
54. Sanchez-Lorente, D., et al., *Prehabilitation in thoracic surgery.* Journal of Thoracic Disease, 2018. **10**(Supplement22): p. S2593-S2600 DOI: http://dx.doi.org/10.21037/jtd.2018.08.18.
55. Sebio Garcia, R., et al., *Functional and postoperative outcomes after preoperative exercise training in patients with lung cancer: a systematic review and meta-analysis.* Interactive cardiovascular and thoracic surgery, 2016. **23**(3): p. 486-97 DOI: https://dx.doi.org/10.1093/icvts/ivw152.
56. Singh, F., et al., *A systematic review of pre-surgical exercise intervention studies with cancer patients.* Surgical Oncology 2013 Jun;22(2):92-104, 2013.
57. Song, S., et al., *Ameliorative effects of Tai Chi on cancer-related fatigue: a meta-analysis of randomized controlled trials.* Supportive Care in Cancer 2018 Jul;26(7):2091-2102, 2018.
58. Song, Y., et al., *Current Evidence on Traditional Chinese Exercise for Cancers: A Systematic Review of Randomized Controlled Trials.* International journal of environmental research and public health, 2020. **17**(14) DOI: https://dx.doi.org/10.3390/ijerph17145011.
59. Stoner, L., et al., *Exercise training, circulating cytokine levels and immune function in cancer survivors: A meta-analysis.* Brain, Behavior, and Immunity, 2019. **81**: p. 92-104 DOI: http://dx.doi.org/10.1016/j.bbi.2019.08.187.
60. Tahirah, F., et al., *Exercise training for people following lung resection for non-small cell lung cancer (NSCLC)-a cochrane systematic review.* Respirology, 2014. **19**(SUPPL. 2): p. 66 DOI: http://dx.doi.org/10.1111/resp.12263.
61. Thompson, E., I. Sola, and M. Subirana, *Non-invasive interventions for improving well-being and quality of life in patients with lung cancer -- a systematic review of the evidence.* Lung Cancer 2005 Nov;50(2):163-176, 2005.
62. Turner, R.R., et al., *Interventions for promoting habitual exercise in people living with and beyond cancer.* The Cochrane database of systematic reviews, 2018. **9**: p. CD010192 DOI: https://dx.doi.org/10.1002/14651858.CD010192.pub3.
63. Wang, Y.-Q., et al., *Effects of Home-Based Exercise Training for Patients With Lung Cancer.* Oncology nursing forum, 2019. **46**(4): p. E119-E134 DOI: https://dx.doi.org/10.1188/19.ONF.E119-E134.
64. Yorke, J., A. Brettle, and A. Molassiotis, *Nonpharmacological interventions for managing respiratory symptoms in lung cancer.* Chronic respiratory disease, 2012. **9**(2): p. 117-29 DOI: https://dx.doi.org/10.1177/1479972312441632.
65. Zhao, I. and P. Yates, *Non-pharmacological interventions for breathlessness management in patients with lung cancer: a systematic review.* Palliative medicine, 2008. **22**(6): p. 693-701 DOI: https://dx.doi.org/10.1177/0269216308095024.
66. Zhao, Y., et al., *Systematic review and meta-analysis on perioperative intervention to prevent postoperative atelectasis complications after thoracic surgery.* Annals of palliative medicine, 2021. **10**(10): p. 10726-10734 DOI: https://dx.doi.org/10.21037/apm-21-2441.
67. Zhou, L., Q. Chen, and J. Zhang, *Effect of Exercise on Fatigue in Patients with Lung Cancer: A Systematic Review and Meta-Analysis of Randomized Trials.* Journal of palliative medicine, 2021 DOI: https://dx.doi.org/10.1089/jpm.2020.0504.

68. Cavalheri, V. and C. Granger, *Preoperative exercise training for patients with non-small cell lung cancer.* Cochrane Database Syst Rev, 2017. **6**: p. CD012020 DOI: 10.1002/14651858.CD012020.pub2.
69. Rosero, I.D., et al., *Systematic Review and Meta-Analysis of Randomized, Controlled Trials on Preoperative Physical Exercise Interventions in Patients with Non-Small-Cell Lung Cancer.* Cancers, 2019. **11**(7): p. 944-944 DOI: 10.3390/cancers11070944.
70. Li, X., et al., *Impact of preoperative exercise therapy on surgical outcomes in lung cancer patients with or without COPD: a systematic review and meta-analysis.* Cancer management and research, 2019. **11**: p. 1765-1777 DOI: https://dx.doi.org/10.2147/CMAR.S186432.
71. Pu, C.Y., et al., *Effects of Preoperative Breathing Exercise on Postoperative Outcomes for Lung Cancer Patients Undergoing Curative Intent Lung Resection: A Meta-Analysis.* Arch Phys Med Rehabil, 2021 DOI: 10.1016/j.apmr.2021.03.028.
72. Gravier, F.A.-O., et al., *Effects of exercise training in people with non-small cell lung cancer before lung resection: a systematic review and meta-analysis. LID - thoraxjnl-2021-217242 [pii] LID - 10.1136/thoraxjnl-2021-217242 [doi].* Thorax, 2021 DOI: 10.1136/thoraxjnl-2021-217242.
73. de Oliveira Vacchi, C.A.-O., B.A.-O.X. Martha, and F.A.-O. Macagnan, *Effect of inspiratory muscle training associated or not to physical rehabilitation in preoperative anatomic pulmonary resection: a systematic review and meta-analysis.* Support Care Cancer, 2021. **30**(2): p. 1079-1092 DOI: 10.1007/s00520-021-06467-4.
74. Li, J., et al., *Effects of exercise training on patients with lung cancer who underwent lung resection: A meta-analysis.* World Journal of Surgical Oncology, 2017. **15**(1): p. 158 DOI: http://dx.doi.org/10.1186/s12957-017-1233-1.
75. Sommer, M.S., et al., *Effect of postsurgical rehabilitation programmes in patients operated for lung cancer: A systematic review and meta-analysis.* Journal of rehabilitation medicine, 2018. **50**(3): p. 236-245 DOI: https://dx.doi.org/10.2340/16501977-2292.
76. Cavalheri, V., et al., *Exercise training undertaken by people within 12 months of lung resection for non-small cell lung cancer.* Cochrane Database of Systematic Reviews, 2019. **2019**(6): p. CD009955 DOI: http://dx.doi.org/10.1002/14651858.CD009955.pub3.
77. Larsen, K.S., et al., *The effects of various respiratory physiotherapies after lung resection: a systematic review.* Physiotherapy Theory and Practice, 2020. **36**(11): p. 1201-1219 DOI: 10.1080/09593985.2018.1564095.
78. Wang, Y.Q., et al., *Impact of breathing exercises in subjects with lung cancer undergoing surgical resection: A systematic review and meta-analysis.* Journal of Clinical Nursing (John Wiley & Sons, Inc.), 2018. **28**(5/6): p. 717-732 DOI: 10.1111/jocn.14696.
79. Machado, P., et al., *Effect of Exercise Training on Quality of Life after Colorectal and Lung Cancer Surgery: A Meta-Analysis.* Cancers (Basel), 2021. **13**(19) DOI: 10.3390/cancers13194975.
80. Xu, X.A.-O., et al., *The effectiveness of pre- and post-operative rehabilitation for lung cancer: A systematic review and meta-analysis on postoperative pulmonary complications and length of hospital stay.* Clin Rehabil, 2022. **36**(2): p. 172-189 DOI: 10.1177/02692155211043267.
81. Liu, X., Y.-Q. Wang, and J. Xie, *Effects of Breathing Exercises on Patients With Lung Cancer.* Oncology nursing forum, 2019. **46**(3): p. 303-317 DOI: https://dx.doi.org/10.1188/19.ONF.303-317.
82. Zhou, L., Q. Chen, and J. Zhang, *Effect of Exercise on Fatigue in Patients with Lung Cancer: A Systematic Review and Meta-Analysis of Randomized Trials.* J Palliat Med, 2021. **24**(6): p. 932-943 DOI: 10.1089/jpm.2020.0504.
83. Heredia-Ciuró, A., et al., *High-intensity interval training effects in cardiorespiratory fitness of lung cancer survivors: a systematic review and meta-analysis.* Support Care Cancer, 2022. **30**(4): p. 3017-3027 DOI: 10.1007/s00520-021-06647-2.
84. Singh, B., et al., *Exercise for Individuals With Lung Cancer: A Systematic Review and Meta-Analysis of Adverse Events, Feasibility, and Effectiveness.* Semin Oncol Nurs, 2020. **36**(5): p. 151076 DOI: 10.1016/j.soncn.2020.151076.

85. Peddle-McIntyre, C.J., et al., *Exercise training for advanced lung cancer.* Cochrane Database of Systematic Reviews, 2019. **2019**(2): p. CD012685 DOI: http://dx.doi.org/10.1002/14651858.CD012685.pub2.
86. Lee, J., *Physiologic and psychologic adaptation to exercise interventions in lung cancer patients undergoing chemotherapy: a systematic review and meta-analysis of randomized controlled trials.* Supportive Care in Cancer, 2021. **29**(6): p. 2863-2873 DOI: http://dx.doi.org/10.1007/s00520-020-05939-3.
